# From Abstinence to Relapse: A Preliminary Qualitative Study of Drug Users in a Compulsory Drug Rehabilitation Center in Changsha, China

**DOI:** 10.1371/journal.pone.0130711

**Published:** 2015-06-24

**Authors:** Mei Yang, Jules Mamy, Pengcheng Gao, Shuiyuan Xiao

**Affiliations:** 1 Department of Social Medicine and Health Management, School of Public Health, Central South University, Changsha, China; 2 Shenzhen Kangning Hospital, Shenzhen Mental Health Center, Shenzhen, China; 3 Hunan Judicial Police Vocational College, Changsha, China; Peking University, CHINA

## Abstract

**Background:**

Relapse among abstinent drug users is normal. Several factors are related to relapse, but it remains unclear what individuals’ actual life circumstances are during periods of abstinence, and how these circumstances facilitate or prevent relapse.

**Objective:**

To illuminate drug users’ experiences during abstinence periods and explore the real-life catalysts and inhibitors contributing to drug use relapse.

**Method:**

Qualitative in-depth interviews were conducted with 20 drug users recruited from a compulsory isolated drug rehabilitation center in Changsha. The interviews were guided by open-ended questions on individuals’ experiences in drug use initiation, getting addicted, treatment history, social environment, abstinence, and relapse. Participants were also encouraged to share their own stories. Interviews were digitally recorded and fully transcribed. The data of 18 participants who reported abstinence experiences before admission were included in the analyses. The data were analyzed using a thematic analysis with inductive hand coding to derive themes.

**Results:**

Most drug users were able to successfully abstain from drugs. During abstinence, their lives were congested with challenges, such as adverse socioeconomic conditions, poor family/social support, interpersonal conflicts, and stigma and discrimination, all of which kept them excluded from mainstream society. Furthermore, the police’s system of ID card registration, which identifies individuals as drug users, worsened already grave situations. Relapse triggers reported by the participants focused mainly on negative feelings, interpersonal conflicts, and stressful events. Craving was experienced but not perceived as a relapse trigger by most participants.

**Conclusions:**

This study of in-depth interview with drug users found evidence of situations and environments they live during abstinence appear rather disadvantaged, making it extremely difficult for them to remain abstinent. Comprehensive programs on relapse prevention that acknowledge these disadvantages are implicated.

## Introduction

Drug addiction has severe medical, legal, social, and economic consequences, and currently is a major problem in China [1–3]. By the end of April 2014, about 2.58 million drug users were officially registered in China, but the estimated actual number may exceed 10 million [3]. In China, heroin is the most common abused drug, with around 53% of all registered drug users reporting heroin use, followed by methamphetamine and ketamine, which have spread quickly in China recent years and account for about 45% of drug users [3].

Drug addiction has been conceptualized as a chronic relapsing condition wherein a relapse is defined as when a person returns to using a drug for which they had previously established abstinence [4, 5]. Much like with other chronic psychiatric conditions (e.g., affective disorders, schizophrenia), relapse prevention is crucial for the control of substance use disorders [6]. The therapeutic process for substance use disorders should optimally begin with detoxification, followed by rehabilitation and relapse prevention utilizing medication and behavioral therapy, alone or in combination [7, 8]. Unfortunately, despite every effort to the contrary, rates of relapse following withdrawal of a treatment are currently very high [8, 9]. In China, relapse rates for drug use after discharge from treatment were generally above 90% within one year [9]. As such, it would be both highly important and urgent to develop and implement better relapse prevention strategies for drug-addicted populations.

Understanding the factors relating to relapse provides basic information for the development of relapse prevention strategies. Indeed, some prior studies have looked at a number of psychosocial and biological factors assumed to predict relapse. Psychosocial risk factors include interpersonal pressures such as peer pressure [10]; adverse socioeconomic conditions, such as low literacy, unemployment, lack of housing, and social and neighborhood problems; and lack of support, whether that support is spiritual, material, or cultural, with emphases on isolation and lack of recreation, trust, and social insurance [11–16]. Craving might also be a strong predictor of relapse [17, 18], and many current therapies for addiction focus on reducing or managing substance craving [19, 20].

However, thus far, most of these prior studies were quantitative, having examined or tested risk factors conceived by researchers before the studies were designed or implemented. The real-life circumstances of drug-addicted individuals and their experiences within these circumstances remain unclear; this makes it difficult to understand exactly what facilitates or prevents relapse based on these individuals’ lived experiences. Understanding the real lives of drug-addicted individuals is of great significance for tailoring interventions to such individuals, thereby making them more pragmatic and accessible. Qualitative studies provide insights into what individuals’ reality is like in a certain natural context, how the individuals experience and respond to that reality, and why they suffer with it; though they do not provide a representation of participants as surveys do. Thus, we conducted a qualitative study using in-depth interviews with drug users to reveal what their lives are truly like. Our focus was on illuminating their experiences during abstinence periods, as this would help us understand the real-life catalysts—and possible inhibitors—of drug use relapse.

Currently, compulsory isolated rehabilitation is a major drug rehabilitation modality in China. According to Chinese drug law, drug addicts who have refused to receive community-based rehabilitation or have failed to maintain abstinence in community, or who have been arrested for having a severe drug addiction would be sent for 1–3 years to compulsory isolated rehabilitation centers managed by the Ministry of Justice [21, 22]. As under these conditions of admission, drug users admitted to compulsory isolated rehabilitation centers might have more severe addictions than those living in communities might. In 2013, about 242,000 drug-addicted individuals were sent to compulsory isolated rehabilitation centers [23]. Given this huge number and the possibility that individuals in these centers have more severe addictions on drugs, this qualitative study was conducted in a compulsory isolated drug rehabilitation center, and we selected purposively a center with a high admission rate in Changsha, China.

## Method

### Participants

We recruited a purposive sample of participants with drug dependence from drug users incarcerated in the Hunan Xinkaipu Compulsory Isolated Drug Rehabilitation Center (XCIR) located in Changsha, China. This is a compulsory rehabilitation institution for males only, which accommodates over 1,000 individuals who engage in drug use [24]. Recruitment was performed by a psychological counselor working at the XCIR, who invited individuals that were potentially eligible for the study to participate. The eligibility criteria were as follows: (1) diagnosed with drug dependence, (2) aged≥18 years, (3) no language difficulties, and (4) no severe mental health problems. Participants’ diagnoses were first provided by the information system for admitters recorded and managed by the XCIR staff, then further judged and confirmed by the interviewer (the first author, a psychiatrist specializing in drug abuse treatment) according to the DSM-IV diagnostic system during the in-depth interview with participants. The number of participants was determined using a theoretical sampling technique (recruiting was halted when little new data emerged) [25]. Finally, 20 participants with drug dependence were recruited and completed the in-depth interview. Of the 20 participants, 18 reported having a history of abstinence before admission (abstinence, as defined in this study, was living an actively sober life by abstaining from illicit drugs after detoxification from these drugs and not in controlled environments), whereas two participants denied any such history. We excluded the two who reported having no abstinence history from the analyses in order to adhere to the study purpose. All 18 participants were male, with a mean age of 33 years (range 18–41 years); length of drug use was, on average, 12 years (range 3–19 years), while the time since admission to the center ranged from 1 to 19 months. Half of the participants (9 of 18) were unemployed before admission and more than half (11 of 18) were below or at the education level of junior high school (see Table 1).

**Table 1:** Characteristics of participants.

Participant No.	Gender	Age	Drug of choice	Years of drug use	Times of abstinence attempt	Environments during abstinence	Months since admission	Employment	Education level	Marital status
1	male	31	heroin	10	many, not mentioned exactly	home or away from home	7	unemployed	junior high school	cohabited
2	male	31	heroin and methamphetamine	11	1	home	12	employed	high school	unmarried
3	male	34	heroin	8	several, not mentioned exactly	away from home	13	unemployed	primary school	married
4	male	39	heroin	15	not mentioned exactly	away from home	17	employed	primary school	divorced
5	male	18	methamphetamine	3	1	in the local township	5	unemployed	junior high school	cohabited
6	male	24	methamphetamine	8	many, not mentioned exactly	home or away from home	1	employed	junior high school	unmarried
7	male	34	methamphetamine	18	2	home	9	employed	junior high school	unmarried
8	male	40	heroin and buprenorphine	16	1	home	15	unemployed	high school	unmarried
9	male	31	heroin and methamphetamine	16	4	home or away from home	16	employed	junior high school	divorced
10	male	36	heroin and methamphetamine	19	2	home	1	employed	junior high school	cohabited
11	male	41	heroin	19	2	home	15	employed	junior high school	married
12	male	40	heroin	11	many, not mentioned exactly	away from home	17	unemployed	high school	divorced
13	male	34	heroin	16	several, not mentioned exactly	away from home	17	unemployed	high school	divorced
14	male	32	heroin and methamphetamine	16	2	home or away from home	14	employed	junior high school	unmarried
15	male	33	heroin and methamphetamine	8	2	home	7	employed	high school	divorced
16	male	23	ketamine and methamphetamine	6	1	away from home	18	unemployed	high school	unmarried
17	male	40	heroin	17	1	home	19	unemployed	high school	unmarried
18	male	33	methamphetamine	3	1	home	7	unemployed	junior high school	divorced

### Interviewer

A face-to-face in-depth interview was performed for each participant during the study. The first author conducted all the interviews with participants. A psychological counselor with a master degree in psychology worked as an on-site assistant for the interviewer and posed some supplementary questions to interviewees. Both the interviewer and the assistant had received training in basic approaches to qualitative research and how to conduct narrative interviews. The training involved a general introduction to qualitative research and its distinction from quantitative methods; interviewing skills such as establishing a rapport, using empathy, gaining trust, avoiding leading questions or imposing one’s own judgment or opinions, and appropriately using emotion; and adherence to professional and ethical boundaries. Furthermore, the training involved role-playing on dealing with difficult occasions, ensuring safety, and giving feedback to interviewees.

### Procedure

The recruitment and interviews were conducted from August through October 2013. Interviews were conducted in a private setting and participants were assured that they could end the interview at any time or decline to answer any question(s) without consequence. All participants signed an informed consent form before the interview began. The interviews followed a topic guide developed by the authors and pilot-tested with four drug users before the interviews were formally conducted. The guide covered five domains, as follows:

- History of drug use initiation
- History of losing autonomous control of drug use and becoming dependent on drugs
- Treatment history and social environment information such as friends and family
- History of abstinence attempts and experiences between start of abstinence and relapse
- Process on this current admission

Participants were also encouraged to share their own stories and engage in meaningful conversation with the interviewer. Interviews were conducted in Mandarin Chinese, and each lasted for 1 to 3 hours. With permission from the participants, the interviews were digitally recorded. The digital audio records were then fully transcribed in Chinese for data analysis with participants anonymized and numbered to preserve their confidentiality. Each participant was given a small gift valued at around 15 Chinese yuan (one Chinese yuan = 0.1626 US dollars) in appreciation for their time. The study procedures and procedures to ensure adherence to ethical guidelines and human participant protection were reviewed and approved by the research ethics committee of the School of Public Health at Central South University.

### Analyses

The data of the in-depth interviews were analyzed using thematic analysis. Thematic analysis is a method for identifying, analyzing, and reporting themes within the data [26]—these themes capture something important about the data in relation to the research question, representing some level of patterned response or meaning within the data set. It is different from grounded theory in that grounded theory requires the analysis to be directed towards theory development (i.e., generate a theory from the data) while thematic analysis does not [26]. Thematic analysis in this study followed the five-step process outlined by Braun and Clarke [26]: (1) familiarization with data; (2) generation of initial codes; (3) searching for themes; (4)review of themes; and (5) definition and naming of themes. These steps are not unidirectional, but rather are continuously repeated as needed during the analysis process.

An inductive hand coding process was employed to derive the themes. Initially, open coding was performed. Open coding involves breaking down the transcripts into component data units consisting of single quotations. These data units were then summarized and assigned a concept by the researchers, and codes were developed and named according the data units’ concepts. Then, axial coding was performed to derive the final themes from the data. During axial coding, codes with similar concepts were grouped together into subcategories, which were in turn organized into categories by making connections between subcategories; this process resulted in the creation of subthemes and themes, which were based on the various subcategories and categories, respectively. The coding procedure was conducted by the first author and the on-site assistant (the third author) independently; then, both were reviewed and judged by the first author according to her expertise. In addition, the authors repeatedly read the transcripts to identify key themes corresponding to the interview guide and emergent topics, and to identify participants’ key statements. Subsequent analyses were undertaken by all authors to examine the consistency of reports across themes and examine any negative evidence. Where inconsistencies in the themes and subthemes were found, the authors engaged in discussions until a consensus was reached. The analysis included data from all parts of the interview, but we focused on participants’ experiences of abstinence to relapse, given the purpose of the current study.

## Results

Three themes relevant to the purpose of this study were identified: (1) ways of overcoming withdrawal and the driving force for abstinence, (2) experiences during periods of abstinence, and (3) “why I relapsed.” For more details, see the appendix (i.e., “summary of themes and subthemes”).

### Ways of overcoming withdrawal and the driving force for abstinence

All 18 participants reported periods of abstinence during their drug use careers, with durations from about 2 months to more than 10 years. They described how they overcame withdrawal symptoms and what the driving forces for their abstinence were.

#### Ways of overcoming withdrawal

Participants described the various ways that they overcame withdrawal symptoms: leaving their hometown (four participants); utilizing medicines/other drugs (two participants); concentrating on work (two participants); tapering off methadone doses (one participant); participating in methadone maintenance treatment (MMT) (one participant); detoxification in compulsory rehabilitation settings (four participants); or utilizing only their own willpower (nine participants). A majority of interviewees expressed that it was actually not very difficult to overcome the physical withdrawal and get their bodies clean.

*“I don’t think it’s very difficult; I can control it*. *If I think I’d be too addicted*, *I’d leave town [and] go down to the village for a period of time*, *a dozen days [or] one month or two*, *to overcome the withdrawal symptoms and get the body clean…the physical addiction is easy to quit*, *only a few days are needed*.*”* (Participant 1)

*“…Finally*, *I tapered the methadone dose down to 2 ml/d*, *and thus got rid of the physical addiction and got off the drugs*.*”* (Participant 2)

*“…I went to a clinic*, *requested some intravenous drips and some tablets*, *and then stayed at home and lay in bed all day long…in this way*, *I quit heroin without suffering much*. *People just never believe*!*”* (Participant 3)

*“You know*, *I tried to quit drugs for about two months using just my willpower in 2002*, *when I had just gotten addicted [to heroin]*. *During the first month [of abstinence]*, *I didn’t eat anything at all*, *I would vomit all the food if I did…”* (Participant 10)

#### Driving force for abstinence

Family responsibility: The majority of interviewees (13 of the 18) described family responsibilities as the main driver for abstinence, with their responsibilities for their children and parents being the most frequently mentioned (six interviewees mentioned the former on 16 occasions and nine interviewees mentioned the latter on 14 occasions).

*“You know what*? *To care for my mother*, *I participated in MMT and stayed drug free for five years*.*”* (Participant 10)

*“I am divorced*, *with a son between 8 and 9 years old; those drug-free years were for him*. *He is my life*.*”* (Participant 15)

*“My family and many of my relatives and friends were getting drenched because of me; for this reason*, *I decided to start following a good life*, *and not be a drug addict any longer…”* (Participant 11)

Normal life: More than half of the interviewees (11of 18) reported giving up drugs in order to live a normal life.

*“About two months after being released [from a compulsory drug rehabilitation center]*, *I didn’t use drugs*, *as I wanted to start on the right path*.*”* (Participant 9)

*“I thought*, *‘I’m over thirty’*. *Thirty years old*! *~If I continue to forsake the right path and go astray*, *I’ll be stuck in a permanent cringe [i*.*e*., *never get better]*. *With that thought*, *I stayed drug-free in Changsha for half a year*.*”* (Participant 9)

Other driving forces: Some interviewees expressed other driving forces, including building or recovering relationships with friends and relatives (four participants), “not wanting to be looked down upon” (three participants), having responsibility for their friends (one participant), acknowledging that “drugs are harmful to the body” (one participant), and “running out of money” (one participant).

*“I was running out of money*. *I’m timid*. *I don’t dare to steal or rob*. *So I stayed at home and abstained from drugs gradually*. *If I had money*, *I’d still use…”* (Participant 11)

### Experiences during periods of abstinence

#### Socioeconomic conditions

Most participants expressed that they lived in disadvantaged socioeconomic conditions and felt powerless to change—poor education, unemployment, and insufficient income were the most frequently emphasized problems.

Education: Of the 18 participants, 11 reported having only a junior-high-school level of education or below, with one of them having received only two years of primary education. The other seven had finished (six participants) or dropped out of (one participant) high school. Except for one, none of the participants had undergone any skills training programs. Participants expressed that with their low education level and lack of knowledge and skills, it was very difficult to get a satisfying job or even know what jobs they were qualified for.

*“I have little schooling*, *so I don’t know many [Chinese] characters and also don’t know how to write… I lost my family at an early age…I wonder what I can do if I don’t [have a good education]…”* (Participant 3)

*“I had no idea*. *With so little schooling*, *except for hard physical labor*, *I don’t know what to do*. *I had no capital to do business*. *Even if I had*, *I could have lost it*.*”* (Participant 9)

*“My eldest sister got mad because I was failing in school and playing every day*. *She always beat me for that*, *but [that beating] didn’t work*. *Some families don’t have enough money to send children to school*. *I wanted to leave school even though I was in a wealthy family*.*”* (Participant 11)

Employment and income: Half of the participants (9 of 18) reported that they were unemployed, were “globe-trotting troubadours” (i.e., had no job, were idle much of the day, engaged in unproductive activities or play, and were friends with similar individuals), or occasionally took some odd jobs during their abstinence periods. They lived with no steady income. Among them, four participants were dependent on their families or sex partners; one made money by stealing, robbing or other illegal means; one lived on government aid; and three mentioned they earned money by every means without describing the details.

*“Without any job*, *I tried to get money by any means; my girlfriend gave me a lot of money*.*”* (Participant 1)

*“I have no fixed place to live and no permanent work; some days I’m here and some days I’m there*. *It’s very difficult to find a job outside*.*”* (Participant 3)

Among those with full-time jobs during abstinence, one was a peasant, one a migrant worker, one an unskilled laborer, and one a low-waged skilled worker. These four had incomes of less than 20,000 yuan per year, which is considered very low.

*“Being a peasant*, *[I make] several thousand yuan a year*, *so there is never enough money*, *even when I’m abstinent*.*”* (Participant 4)

In contrast to the majority, four participants reported working or doing business and receiving plenty of money.

#### Family/social supports

Family/social support: Most participants (13 of 18) spoke of lacking necessary family/social support during abstinence periods. Over the years, their family ties had been stretched to the limit or broken entirely; estrangement from loved ones was a central theme. They spoke about many aspects of such estrangement, such as being divorced, living alone away from one’s family, being deserted by relatives and friends, lacking family and social ties, living in poverty without insurance, lacking understanding from others and feeling loneliness.

*“I was divorced then*. *Even my relatives and friends deserted me*. *[In this situation] whom could I turn to for help [then*?*] Who would really care you*? *[Nobody*!*]”* (Participant 4)

*“Sometimes*, *when I was in trouble*, *I couldn’t find anyone to talk to about it*.*”* (Participant 6)

*“I had participated in MMT for about five years…but a major obstacle was that my family didn’t understand it [MMT]; they regarded methadone as a drug like heroin*, *so although occasionally my mother would agree with me [that methadone is a good way to maintain a normal life]*, *relatives and friends didn’t understand at all*. *They thought I was still taking drugs and that I was still dependent on them and hopelessly damaged*.*”* (Participant 10)

However, one participant reported having good family support.

*“My family supports me*. *When I wanted to open up a shop*, *they were willing to give me the startup capital*.*”* (Participant 16)

Family conflicts: Two participants described their experiences with family conflicts, which were considerable sources of stress for them; indeed, both said that these conflicts had contributed to their relapse.

*“When my father died*, *my wages weren’t very high*, *only about ten thousand yuan a year; however*, *this ten thousand yuan was always taken away by my mother*. *My mother thinks that all of my family owes her*. *I wonder if she is really my mother or not…She always quarreled with me about money*, *more than anything else*, *saying that I was good for nothing for running out of money and all kinds of crap like that*. *I was so annoyed… So I went back to ice [crystal methamphetamine]*.*”* (Participant 2)

#### Stigma

Stigmatization and discrimination: Participants said that despite their being abstinent—even for many years—they were still looked upon as drug addicts and suffered continual stigmatization. They found it almost impossible to conceal their identities as addicts. Acquaintances, relatives, and even immediate family distrusted and excluded them, and they were often labeled as dishonest or evil and degraded by others, including medical or other service providers.

*“Even when I’d stopped taking drugs*, *people wouldn’t believe it; when we go out into the real world*, *many just discriminate against us*.*”* (Participant 2)

*“People just look down on you; [being] at the bottom of the heap*, *who would look up to us*? *Nobody*!*”* (Participant 4)

*“Acquaintances…turned back when seeing me in their way*, *like [they were] avoiding a monster*.*”* (Participant 8)

*“That old guy*! *He had received so much kindness from me*, *but still feels ashamed of knowing me…He said to me ‘It’s not that I don't want to help you*, *but the others would exclude me if I did so…’ He thinks I’ve had some negative effect on him*.*”* (Participant 17)

*“When I was working in his factory*, *my uncle*, *the boss*, *didn’t trust me*. *He said ‘You needn’t make that excuse to scrounge money off me…’ But I had indeed stopped using drugs*.*”* (Participant 16)

*“I was on the edge of death at that time; twice I was given last rites while lying gravely ill in a hospital*, *having abscesses and sepsis and being paralyzed*. *The hospital in Shenzhen did not accept me so I was transferred to a local hospital*. *But just right after the transfer*, *the local hospital refused me also…At last*, *I was put in a special ward for drug addicts*. *That’s the situation of our hospitalization*. *When we seek medical care or require hospitalization*, *we are refused or*, *if lucky*, *segregated with patients who belong to our kind—drug users*.” (Participant 17)

Self-stigma: Some interviewees referred to a sense of self-stigma, resulting from their feelings of “wrong-doing,” “law-breaking,” “deviance,” and “inferiority,” and from the verbal and non-verbal discrimination of others.

*“Drug users have bad characters and are dishonest*, *deceitful*, *and venal*.*”* (Participant 1)

*“Drug users are worthless and hopeless*. *I’d never be able to hold up my head*.*”* (Participant 3)

*“Sometimes—maybe for my own reasons—when I was back at home*, *I felt that it was very difficult to speak to relatives; I bowed my head among them*, *and felt as if I had no friends…”* (Participant 6)

*“Using drugs is more shameful than stealing or even murdering*. *It’s too shameful to get any help*. *I don’t resent people for not helping*, *because drug users do ugly things*, *being the most inferior persons*.*”* (Participant 8)

Outcome of stigma: One outcome of stigmatization for participants was their exclusion from mainstream society, including employment opportunities and friendships with normal people; generally, participants’ friend circles were limited to only other drug users or they were completely isolated. Moreover, participants were often regarded as less deserving groups in competition for limited resources such as subsistence allowances and low-rent housing for low-income populations from the government. Another outcome was their increased likelihood of rejecting community or other public services, whether drug-related or not, considering the lack of privacy in using such services and the discrimination from local residents if recognized. Both outcomes exacerbated participants’ struggles with addiction.

*“I feel inferior*, *with a heart of glass—very fragile and easily hurt—so I dare not hope for a friendship with a non-user; I had to choose and inhabit drug users’ circles or a world consisting only of ‘our kind’*.*”* (Participant 14)

*“Regarding employment*, *there is also social discrimination against us*. *Low-rent housing*? *No way*, *they’d never give it to us*, *they think that we’re not deserving*. *They said with discrimination that drug users are not eligible for subsistence allowances offered by the government…”* (Participant 17)

*“I was not gonna use the community services out here; don’t need everybody in the community knowing that ‘you are an addict’*, *which is a disgrace and shameful…My family wouldn’t go out to blather*.*”* (Participant 1)

*“As a drug user*, *people won’t give you another look*. *No way*! *Anyway*, *to solve loneliness*, *I went looking for something to get numbed…”* (Participant6)

Not all participants expressed feelings of being judged negatively by others. It may be that some users either were not sensitive to such judgments or could not verbalize their sensitivity.

#### ID card registration and police enforcement

Some participants (6 of 18) complained about how the police’s ID card registration system for drug users and the consequent police enforcement disrupted their everyday lives, deterred their access to treatment services such as MMT, held them back from job opportunities, and even led to arbitrary arrests.

*“Occasionally*, *yes*, *I got some methadone from acquaintances who entered MMT*, *but I would not enter it myself because the ID card has to be registered by the police station*. *Once that ID card is registered*, *how can I walk around outside*?*…The network for the ID card is very troublesome*. *If I registered [as a drug user]*, *I’d not even dare to get a hotel room because police would know it as soon as I checked in*.*”* (Participant 1)

*“I had just checked into a hotel room*, *only for about 30 minutes; then*, *somebody knocked the door*. *I thought it was the room attendant*, *but it was the police*! *I was forced to the police station and made to do a urine test…It was my ID card*, *which is linked with the information of me ‘being a drug user’*, *and this alerted the local police as I was checking in…I’m afraid of using the ID card outside*, *let alone using it for job searching*.*”* (Participant 9)

*“MMT is good*, *but a big issue is that police always arrest clients at the door*. *Wherever there is a positive urine testing*, *people would be arrested…They [the policemen] just squat there; If you do something that one of them doesn't like*, *you’ll be somebody who should submit a urine sample…the corrupt cop*!*”* (Participant 10)

#### Urge or desire to use drugs (craving)

More than half of the interviewees (12 of 18) reported experiencing the urge or desire to use drugs while in abstinence.

*“The conscious desire*, *ah*, *I couldn’t quit*, *as long as the physical addiction wasn’t that severe*. *There’s always a desire*, *or rather*, *a hunger [for drugs]*.*”* (Participant 1)

*“I don’t know why…that [craving] could not be eliminated…Anyway*, *the desire [for drugs] is always there*.*”* (Participant 3)

*“I just can't get rid of the craving*. *I know of no way…”* (Participant 4)

Craving happened spontaneously: Sometimes, participants described craving as spontaneously arising, particularly in situations that were troubling or exciting.

*“I had suffered a lot*. *When I got into something troubling*, *I really didn’t know who I could talk to and often the urge to use [drugs] flashed into my mind…”* (Participant 6)

*“Later*, *when in a bad mood*, *I felt a desire to inject*, *even if nobody asked me*.*”* (Participant 11)

*“It was just before our wedding day*. *Maybe because of the joy of the upcoming wedding*, *I felt like I did want to take some drugs…And when I found myself getting upset*, *the craving [for drugs] also arose*. *That’s it*!*”* (Participant 9)

Cue-evoked craving: Participants also emphasized their reactivity to drug-related environmental cues (i.e., cue-evoked cravings). Such cues caused extremely intolerable cravings, which they attempted to suppress with drugs.

*“Whenever I went back home*, *especially into the exact room I had lived in and in which I had always used drugs*, *the urge arose*. *I’m so familiar with the feeling that the drug gives me; it’s such a deep feeling*!*…Often*, *on my return*, *my old friends who used drugs would come over*, *even though I’d not like to see them that soon*. *They called me out to ‘satisfy the craving’*, *and at that moment I felt a surge in craving to go use…”* (Participant 6)

*“I can never go back*! *Once I went back*, *especially when some old friend who used drugs called me*, *I just could not help [thinking of using heroin]*.*”* (Participant 12)

Craving alleviation: Participants reported that cravings diminished or vanished when they changed their place of residence, such as leaving downtown and spending some days in the country, or working and living in another city far away from home.

*“But if I was away from home*, *going from Yueyang city [a city in Hunan province] to*, *for example*, *the country*, *it [the craving] would go away*. *I wonder why*, *but it’s beyond me…And in 2005*, *when I was working [as a migrant worker] with my wife in Panyu [a city in Guangdong province]*, *there was a drug trafficker living in the apartment above ours*, *but I didn’t want to have anything to do with him; in other words*, *I didn’t want any drugs at all*.*”* (Participant 12)

One participant reported changing their pastime in a way that was useful for alleviating the craving.

*“When I felt it [craving]*, *I tried getting some friends*, *into a tea house*, *chatting together for a while*, *or playing Mahjong*. *That doesn't cost much and I would be much better*.*”* (Participant 11)

### “Why I relapse”

During the interviews, 11 of the participants gave descriptions of why they relapsed, which involved aspects of the surrounding circumstances, events, and feelings.

#### “Conscious desire”

Two of them expressed “conscious desire” as a major reason.

*“The desire was always there*, *until I lost control*.*”* (Participant 1)

*“It was very difficult for me to stop the craving*, *which lead to a lapse and then to a relapse*.*”* (Participant 4)

Unexpectedly, the other nine did not mention any cravings to use drugs when describing their reasons for relapse. Rather, the relapse triggers reported by these participants focused mainly on negative feelings, interpersonal conflicts, and stressful events.

#### Negative feeling

The negative feelings governing relapse included feelings of loneliness, emptiness, helplessness, hopelessness, and extreme desperation due to social isolation, social exclusion, and lack of support.

*“Being alone outside*, *I felt really down*, *so I smoked a little dope*.*”* (Participant 3)

*“There was no way out of the loneliness*! *I solved it with drugs… Anyway*, *to get through the feeling*, *you numb [yourself]*!*”* (Participant 6)

*“When I felt confusion or emptiness inside*, *I thought drugs were an escape*. *That’s why I relapsed*.*”* (Participant 15)

*“It's just that I was too lonely at that time*. *I wanted to seek work*, *but having poor health and being a drug user*, *I was actually excluded from every job opportunity*. *I’d been cut off*, *with no source of income*, *no money*, *and no family*. *I was so desperate that I escaped back into drugs…When [people] looked at me*, *their eyes were full of contempt and distrust*. *That hurt me too much…I could only numb myself with drugs*!*”* (Participant 17)

#### Interpersonal conflict

Interpersonal conflicts with family members (e.g., mother) were another main reason for relapse.

*“I don’t know why I relapsed*. *I re-used ice [crystal meth] just because of my mother*. *She squeezed me too much*, *always scolded me*, *and turned all of my arrangements upside down…I hated seeing her*, *particular when seeing her chatting with her own mother… She put too much pressure on me to get off drugs*!*”* (Participant 2)

#### Stressful event

Stressful events—such as divorce or being shamed by others—provoked negative emotional responses, which in turn led to relapse.

*“I might not have relapsed at that time if my wife hadn’t divorced me*.*”* (Participant 9)

*“I relapsed to using ice because my girlfriend broke up with me…and because my friend laughed at me*. *I helped him by getting directly involved in fights*, *but when I was arrested and put in detention for him*, *he just only laughed at me*, *which made me feel pretty upset*!*”* (Participant 16)

Exceptionally, one participant stated that his relapse into ice was due to working irregular hours.

*“We worked in three shifts around the clock*. *When I was too sleepy to stay upon the night shift*, *I would smoke a little ice*.*”* (Participant 14)

Finally, one mentioned that one instance of relapse was due to *“the joy of the upcoming wedding”* (Participant 9).

## Discussion

To our knowledge, this is the first study, both in China and globally, to qualitatively depict drug users’ experiences during periods of abstinence. We found that most drug users had successfully abstained at some point from drugs and had maintained that abstinence for at least for several months. Most had their own ways of overcoming withdrawal symptoms effectively, and mentioned that was not so difficult if they were driven by family responsibility or the longing for a normal life. However, during abstinence periods, their lives were congested with challenges: with little education and job skills, they commonly lived in disadvantaged socioeconomic conditions and were incapable of changing their adverse situations; their family/social supportive systems were poor or were even completely lacking, and sometimes were disturbed by family conflicts; stigma and discrimination were as prevalent to them as air, even after abstaining for many years, and such stigma excluded them from broader social worlds, including job opportunities, normal friend circles, medical services, and even essential living support for the poor provided by the government; and because their ID cards identified them as drug users, they experienced “police phobia” and found their lives severely restricted. Because previous quantitative studies have found these challenges to be risk factors of relapse [11–16, 27], we can conclude that it is extremely difficult for persons with drug use disorders to remain abstinent when living in an environment so redolent with risk factors.

Dialectic philosophy holds that “external causes are the condition of change and internal causes are the basis of change, and that external causes become operative through internal causes [28]. According to this viewpoint, in cases that where there is an internal cause for an individual to recover (e.g., a desire to be responsible for their family, a longing for living a “normal life,” and thoughts of harm from drug use), a change in their environment would likely facilitate internal changes. The Anti-Drug Law of China is now calling for a boost in “the role of communities and families” in helping to reduce drug users’ dependency [21, 22, 29, 30]. However, according to our study, the current situation of the drug users’ environments seems contrary to this goal—they are being excluded from society, and are given little opportunity to excel even after many years of abstinence. Given that relapse prevention programs for drug-addicted patients who have received abstinence-oriented treatment are actually almost totally lacking in China [7], our results suggest an need to develop comprehensive programs aimed at relapse prevention that facilitate recovery in China. More governmental sectors and social organizations should be involved in the creation of a good environment that creates job opportunities, promotes literacy, and provides vocational training and social and family support systems, and eliminates stigma among individuals who have ever used drugs. Furthermore, we recommend that the police’s system of ID card registration for drug users should be deactivated, because it exacerbates stigmatization, limits individuals’ everyday lives, and is being perceived as an obstacle to seeking treatment.

Another important finding of this study that has implications for intervention is that although drug craving was commonly experienced by participants during abstinence, and arose in varied forms (e.g., spontaneously or cue-evoked), most participants did not perceive craving to be a relapse trigger. Indeed, some of them even reported effective ways of coping with cravings. Relapse triggers reported mainly by the participants were negative feelings (e.g., loneliness, helplessness, and hopelessness) in adverse situations (e.g., social isolation, social exclusion, and lacks of support), and interpersonal conflicts and stressful life events. These findings, however, conflict with traditional views and a body of prior quantitative research that indicate craving is a strong causal factor in individuals’ relapse [17–20]. Considering that there were still two participants who reported “conscious desire” as a major relapse reason, and that our findings were drawn from a qualitative study with only 18 participants, it seems too early to draw any conclusions or propose a hypothesis that the studied contextual factors are more important to relapse than are cravings. Hypothesis development should be based on triangulated qualitative studies, and large sample quantitative studies should be designed and conducted before making any conclusions. However, if this is the case, then mainstream relapse programs should shift their focus from reducing cravings to improving individuals’ living conditions and coping skills.

A major limitation of this study was that we used a purposive sample with a limited number of participants recruited from one compulsory drug rehabilitation setting. As such, the findings cannot be generalized to other groups of drug users or to other settings, especially to those living at home, who may not be in such dire situations.

Another limitation is that all the interviews were conducted with drug users; no other informants or stakeholders were recruited, meaning that participants’ reports could have been intentionally dishonest or affected by recall bias (problems common to all studies relying on self-reports). In addition, the validity of drug users’ stories might be compromised by the effects of chronic drug use.

A third limitation is a lack of comparison of the method of this study with previously known qualitative work on this topic, and the method was not triangulated using multiple methods of data collection such as extensive observations.

Despite these limitations, the current study provides initial information on drug users’ experiences during abstinence in China, and we are confident in its validity for the following reasons. First, the data collection was considered rich and in-depth, despite the small number of participants with only 18 included, given that the final decision on sample size was based on the data saturation point and the long duration of 1–3 hours for each interview. Second, interviewer triangulation was supported by using two trained interviewers in each interview, and the fact that the main interviewer was a psychiatrist in the field of drug abuse likely minimized the infiltration of researchers’ values. Third, an inductive coding approach combined with authors’ discussion of the themes developing in the analysis improved the accuracy of the data interpretation. Finally, the social issues raised in our study (e.g., stigma) were in general consistent with the literature relating to drug users [31–33]. Whereas for all of the limitations in the present study, our future study should attempt to involve more diverse participants, including drug users at other situations (community or voluntary settings), key informants or stakeholders to improve the validity of participants’ narratives and derive more convincing findings.

## Appendix

### Summary of themes and subthemes

1. **Ways of overcoming withdrawal and the driving force for abstinence**
  - **Ways of overcoming withdrawal**
  - **Driving force for abstinence**
    - Family responsibility
    - Normal life
    - Other driving forces
2. **Experiences during periods of abstinence**
  - **Socioeconomic conditions**
    - Education
    - Employment and income
  - **Family/social supports**
    - Family/social support
    - Family conflicts
  - **Stigma**
    - Stigmatization and discrimination
    - Self-stigma
    - Outcome of stigma
  - **ID card registration and police enforcement**
  - **Urge or desire to use drugs (craving)**
    - Craving happened spontaneously
    - Cue-evoked craving
    - Craving alleviation
3. **“Why I relapse”**
  - **Conscious desire**
  - **Negative feeling**
  - **Interpersonal conflict**
  - **Stressful event**

## Supporting Information

S1 File Transcripts of words talked by the 18 participants. (DOC)

## References

[1] Li J, Ha TH, Zhang C, Liu H (2010) The Chinese government's response to drug use and HIV/AIDS: a review of policies and programs. Harm Reduct J 7: 4 10.1186/1477-7517-7-4 20205729 PMC2847555

[2] Lu L, Fang Y, Wang X (2008) Drug abuse in China: past, present and future. Cell Mol Neurobiol 28: 479–490. 17990098 10.1007/s10571-007-9225-2 PMC11515005

[3] Yu M (2014) There 2.58 million drug users were officially registered in China, with an annual rate of increase of 36% [in Chinese]. August 24. http://sc.people.com.cn/n/2014/0824/c345167-22085146.html. Accessed 08 September 2014; Available: http://www.webcitation.org/6TFGnZxZA Accessed 11 October 2014.

[4] McLellan AT, Lewis DC, O’Brien CP, Kleber HD (2000) Drug dependence, a chronic medical illness: implications for treatment, insurance, and outcomes evaluation. JAMA 284:1689–1695. 11015800 10.1001/jama.284.13.1689

[5] McLellan AT, McKay JR, Forman R, Cacciola J, Kemp J (2005) Reconsidering the evaluation of addiction treatment: From retrospective follow-up to concurrent recovery monitoring. Addiction 100: 447–458. 15784059 10.1111/j.1360-0443.2005.01012.x

[6] Witkiewitz K, Marlatt GA (2004) Relapse prevention for alcohol and drug problems: That was Zen, this is Tao. American Psychologist 59: 224–235. 15149263 10.1037/0003-066X.59.4.224

[7] Min Z, Xu L, Chen H, Ding X, Yi Z, Mingyuang Z (2011) A pilot assessment of relapse prevention for heroin addicts in a Chinese rehabilitation center. Am J Drug Alcohol Abuse 37: 141–147. 10.3109/00952990.2010.538943 21438799 PMC3113608

[8] James RK, Gilliland BE (2013) Crisis Intervention Strategies. Brooks/Cole Publishing Company pp. 399–403.

[9] Liu ZM (2004) Several suggestions on drug rehabilitation through labor [in Chinese].Chin J Drug Depend 13: 233–234.

[10] Maehira Y, Chowdhury EI, Reza M, Drahozal R, Gayen TK, Masud I, et al (2013) Factors associated with relapse into drug use among male and female attendees of a three-month drug detoxification-rehabilitation programme in Dhaka, Bangladesh: a prospective cohort study. Harm Reduct J 10: 14 (page 1–13 of 13). 10.1186/1477-7517-10-14 24004685 PMC3846454

[11] Sau M, Mukherjee A, Manna N, Sanyal S (2013) Sociodemographic and substance use correlates of repeated relapse among patients presenting for relapse treatment at an addiction treatment center in Kolkata, India. Afr Health Sci 13:791–799. 10.4314/ahs.v13i3.39 24250323 PMC3824454

[12] Henkel D (2011) Unemployment and substance use: a review of the literature (1990–2010). Curr Drug Abuse Rev 4: 4–27. 21466502 10.2174/1874473711104010004

[13] Alverson H, Alverson M, Drake RE (2001) Social patterns of substance use among people with dual diagnoses. Ment Hlth Serv Res 3: 3–14. 10.1023/a:1010104317348 11508560

[14] Quimby E (1995) Homeless clients’ perspectives on recovery in the Washington, DC, dual diagnosis project. Contemporary drug problems summer 265–289.

[15] Sells DJ, Rowe M, Fisk D (2003) Violent victimization of persons with co-occurring psychiatric and substance use disorders. Psych Serv 54:1253–1257. 12954942 10.1176/appi.ps.54.9.1253

[16] Salyers M, Becker DR, Drake RE (2004) A ten year follow-up of supported employment. Psych Serv 55:302–308. 10.1176/appi.ps.55.3.302 15001732

[17] Wray JM, Gass JC, Tiffany ST (2013) A systematic review of the relationships between craving and smoking cessation. Nicotine Tob Res 15:1167–1182. 10.1093/ntr/nts268 23291636 PMC3682845

[18] Stewart J (2008) Review. Psychological and neural mechanisms of relapse. Philos Trans R Soc Lond B Biol Sci 363:3147–3158. 10.1098/rstb.2008.0084 18640921 PMC2607321

[19] Witkiewitz K, Bowen S, Douglas H, Hsu SH (2013) Mindfulness-based relapse prevention for substance craving. Addict Behav 38:1563–1571. 10.1016/j.addbeh.2012.04.001 22534451 PMC3408809

[20] Xue YX, Luo YX, Wu P, Shi HS, Xue LF, Chen C, et al (2013) A memory retrieval-extinction procedure to prevent drug craving and relapse. Science 336:241–245. 10.1126/science.1215070 PMC3695463 22499948

[21] Standing Committee of the National People's Congress (2007) Narcotics Control Law of the People's Republic of China [in China]. Available: http://en.pkulaw.cn/display.aspx?cgid=100676. Accessed 11 October 2014; Archived at: http://www.webcitation.org/6TFHLbmceon Accessed 11 October 2014.

[22] State Council of the People’s Republic of China (2011) Regulation on Drug Rehabilitation [in China]. Beijing: Ministry of Public Security. http://en.pkulaw.cn/display.aspx?cgid=153412. Accessed 11 October 2014; Available: http://www.webcitation.org/6TFHo6oRD. Accessed 11 October 2014.

[23] National Narcotic Control Commission (2014) Annual Report on Drug Control in China. Beijing: Ministry of Public Security.

[24] Yang M, Mamy J, Zhou L, Liao YH, Wang Q, Seewoobudul V, et al (2014) Gender differences in prevalence and correlates of antisocial personality disorder among heroin dependent users in compulsory isolation treatment in China. Addict Behav 39:573–579. 10.1016/j.addbeh.2013.11.003 24342175

[25] Liamputtong P, Ezzy D (2005) Qualitative research methods (2nd ed). Melbourne: Oxford University Press.

[26] Braun V, Clarke V (2006) Using thematic analysis in psychology. Qualitative Research in Psychology 3:77–101.

[27] Meng J, Burris S (2013) The role of the Chinese police in methadone maintenance therapy: A literature review. Int J Drug Policy 24: e25–34. 10.1016/j.drugpo.2013.03.010 23623719

[28] Mao Zedong (2010) On contradiction. Available: http://www.lastsuperpower.net/docs/mao-contradiction. Accessed 9 September 2014; Archived: http://www.webcitation.org/6TFIFzwvjon Accessed 11 October 2014.

[29] State Council of the People’s Republic of China (2011) Regulation on Drug Rehabilitation. Beijing: Ministry of Public Security.

[30] Xinhua News Agency (2013). China issues drug rehabilitation regulation. China Daily. Aug 26, 2013. Available: http://usa.chinadaily.com.cn/china/2011-06/26/content_12777747.htm. Accessed 09 September 2014; Archived: http://www.webcitation.org/6TFJDYmfUon Accessed October 11th, 2014.

[31] Li SP, Zheng ZY, Meng QY, Yuan CH (2013) Barriers to tuberculosis care for drug users in two provinces of China: a qualitative study. Int J Tuberc Lung Dis 17:1358–1363. 10.5588/ijtld.12.0784 24025390

[32] Lin C, Wu Z, Detels R (2011) Opiate users' perceived barriers against attending methadone maintenance therapy: a qualitative study in China. Subst Use Misuse 46: 1190–1198. 10.3109/10826084.2011.561905 21417558 PMC3392119

[33] Luo T, Wang J, Li Y, Wang X, Tan L, Deng Q, et al (2014) Stigmatization of people with drug dependence in China: a community-based study in Hunan province. Drug Alcohol Depend 134: 285–289. 10.1016/j.drugalcdep.2013.10.015 24239068

